# Traumatic Events, Psychopathology, and Post-Traumatic Stress Disorder in the General Community and First Responders: Presence of Symptoms and Associated Factors

**DOI:** 10.3390/ejihpe15120241

**Published:** 2025-11-26

**Authors:** Hélder António, Pedro Gamito, Stéphane Bouchard, Shivani Atul Mansuklal, José Cardoso, Maria Vieira de Castro, Ricardo Pinto

**Affiliations:** 1 HEI-Lab: Digital Human-Environment Interaction Labs, Lusófona University, Campo Grande 376, 1749-024 Lisboa, Portugal; pedro.gamito@ulusofona.pt (P.G.); shivani.atul@ulusofona.pt (S.A.M.); maria.vieira.castro@ulusofona.pt (M.V.d.C.); ricardo.pinto@ulusofona.pt (R.P.); 2 Département de Psychoéducation et de Psychologie, Université du Québec en Outaouais, Gatineau, QC J8X 3X7, Canada; stephane.bouchard@uqo.ca; 3 Departamento de Psicologia da Unidade de Saúde da GNR, Centro Clínico, Rua Presidente Arriaga 9, 1200-771 Lisboa, Portugal; cardoso.jeq@gnr.pt

**Keywords:** PTSD, traumatic events, first responders, general community, psychopathology

## Abstract

Most individuals experience at least one traumatic event during their lifetime, which can lead to the development of psychopathological symptoms and Post-Traumatic Stress Disorder (PTSD). First responders (e.g., police officers, firefighters, emergency medical professionals) are exposed to traumatic events daily, making them particularly vulnerable to developing such symptoms. Using an online questionnaire, this study aimed to compare self-reported exposure to traumatic events and the presence of psychopathological and PTSD symptoms between a sample from the general community (*n* = 137) and first responders (*n* = 672) residing in Portugal. We also sought to identify factors associated with the development of PTSD symptoms. Results showed that although first responders reported higher exposure to traumatic events, there were no significant differences in PTSD symptoms between first responders and the general community. However, general psychopathological symptoms, particularly anxiety and depression, were higher in the general community than among first responders. Symptoms of anxiety, depression, obsessive–compulsive tendencies, hostility, paranoid ideation, psychoticism, and personally experienced traumatic events emerged as significant predictors of PTSD symptoms, whereas demographic variables showed no significant predictive value. The potential influence of factors such as terror management theory, training and education, professional selection, the “hero lifestyle”, and the “police culture” is discussed, along with implications and directions for future research.

## 1. Introduction

Approximately 70% of the general population experiences at least one traumatic event during their lifetime (Benjet et al., 2016; Hyland et al., 2021; Kessler et al., 2017), whereas professionals in high-risk occupations are routinely exposed to potentially traumatic situations (Foley & Massey, 2021; Johnson et al., 2005; Lentz et al., 2021). Traumatic events may include exposure to armed conflict, violent death, or serious accidents involving imminent risk of severe injury or death (Carlier et al., 2000; Mitchell et al., 2017). In professions such as firefighting, law enforcement, and emergency medical services, repeated exposure to such events can adversely affect physical and mental health, increasing the risk of depression, anxiety (Li et al., 2016), and Post-Traumatic Stress Disorder (PTSD) (Marmar et al., 2006; Petrie et al., 2018; Posch & Zube, 2024).

Previous research has shown that the prevalence of PTSD symptoms varies across general populations and high-risk occupational groups (Foley & Massey, 2021). Reported rates of PTSD symptoms range from 1% (Gureje et al., 2006) to 5–9% (Heir et al., 2019), while approximately 6.8% of the general population in the United States experience PTSD symptoms at least once in their lifetime (Gradus et al., 2013). In regions affected by armed conflict, rates can reach up to 37% (De Jong, 2001). Among first responders, prevalence rates differ by sector, ranging from 20% to 30% in emergency medical personnel (Alexander & Klein, 2001; Clohessy & Ehlers, 1999), 14.2% among UK police officers (Syed et al., 2020), 11% among ambulance workers (Petrie et al., 2018), and 13.5% among war veterans (Dursa et al., 2014).

In the Portuguese context, a study conducted with a sample of 2606 participants from the general population reported a 7.87% prevalence of PTSD symptoms, with marked gender differences (11.4% in women vs. 4.8% in men) (De Albuquerque et al., 2003). Among Portuguese first responders, research involving military personnel deployed to Kosovo, Afghanistan, Lebanon, and Timor in 2011 indicated a 22.5% prevalence of PTSD symptoms (Correia, 2015)**,** and another study found that 39% of colonial war veterans exhibited PTSD symptoms (Maia et al., 2011).

Although most people experience at least one traumatic event in their lifetime, not everyone develops PTSD symptoms (Kessler, 1995; Wu et al., 2019). Some may experience transient post-traumatic symptoms that resolve within days or weeks (Adams & Boscarino, 2006; Kessler, 1995). This evidence suggests the existence of multiple etiological pathways linking trauma exposure to PTSD, highlighting the need to identify risk and protective factors associated with the development of PTSD symptoms (Adams & Boscarino, 2006; Martin et al., 2009; McCanlies et al., 2014).

Despite the extensive body of research on PTSD among first responders and the general population, few studies have simultaneously examined both groups within a single design while differentiating between distinct types of trauma exposure as defined by DSM-5 Criterion A (i.e., directly experienced, witnessed, work-related, or heard about). The present study is therefore innovative in its large sample size and its integration of multiple variables, including how traumatic events were experienced, PTSD symptoms, and general psychopathological symptoms.

Accordingly, this study aimed to (1) verify the number of traumatic events among the general community and first responders; (2) examine the relationship between trauma types, PTSD symptoms (SPSSV criteria), and psychological symptoms (BSI) in first responders and the general community; (3) compare the presence of psychopathological symptoms between both groups; (4) compare PTSD symptoms between both groups; and, (5) identify and compare associated factors with PTSD symptoms. This study seeks to contribute to the existing literature by identifying factors associated with PTSD in the context of trauma exposure, thereby informing policymakers and practitioners about the need for prevention, early detection, and intervention strategies in this field.

## 2. Materials and Methods

This study employed a cross-sectional, quantitative design using a self-report questionnaire administered online through the Qualtrics platform.

### 2.1. Participants

A total of 1503 responses were received, of which 694 were excluded due to incomplete questionnaires. The final sample comprised 809 participants aged between 18 and 74 years (*M* = 43.68, *SD* = 9.04). Among these, 137 were from the general community and 672 were first responders (firefighters, police officers, and emergency medical professionals). Most participants were male (*n* = 670, 77.9%), married (*n* = 430, 51.1%), Portuguese (*n* = 800, 98.9%), and had completed secondary education (12th grade). The majority reported living in households of three people (*n* = 249, 30.8%) with a monthly household income between 1000 and 1500 euros (*n* = 206, 25.5%). Additionally, 283 participants (35%) reported having one dependent, 624 (77.1%) reported no health problems, and 525 (64.9%) indicated that they were not taking any medication. Regarding PTSD prevalence, 102 first responders (15.2%) and 18 individuals from the general community (13.1%) met the criteria for PTSD. Additional sociodemographic information is presented in Table 1.

### 2.2. Instruments

Sociodemographic survey questions were developed by the research team to collect information on participants’ age, sex, marital status, nationality, place of residence, educational background, professional activity, years of professional experience, monthly household income, household size, number of dependents, presence of any medical conditions, and current medication use.

Brief Symptom Inventory (BSI) (Derogatis & Melisaratos, 1983) (Portuguese validated version (Canavarro, 2007), which involved translation and psychometric validation for the Portuguese population) is a 53-item self-report instrument that assesses psychopathological symptoms across nine dimensions: Somatization, Obsessive–Compulsive symptoms, Interpersonal Sensitivity, Depression, Anxiety, Hostility, Phobic Anxiety, Paranoid Ideation, and Psychoticism. It also provides three global indices: the Global Severity Index (GSI), which combines symptom intensity and frequency; the Positive Symptom Distress Index (PSDI), which measures the average intensity of reported symptoms; and the Positive Symptom Total (PST), which reflects the total number of symptoms endorsed. Together, these indices offer a comprehensive overview of general psychological distress. Participants rated how much each symptom had distressed or bothered them during the past week using a 5-point Likert scale (0 = Never, 1 = Rarely, 2 = Sometimes, 3 = Often, and 4 = Very often). Mean scores were computed for each dimension, with a cut-off value of 1.7 or higher indicating the presence of clinically relevant symptoms (Canavarro, 2007). In the present study, the BSI demonstrated excellent internal consistency, with a Cronbach’s alpha of 0.98.

Life Events Checklist for DSM-5 ((LEC-5) (Weathers et al., 2013); LEC-5, Portuguese validated version (Carvalho et al., 2020), which involved translation and psychometric validation for the Portuguese population) is a self-report measure designed to screen for exposure to potentially traumatic events and to identify the type of exposure to each event. The instrument consists of 17 items, 16 referring to specific categories of traumatic experiences (e.g., natural disasters, sexual assault, or serious accidents), and 1 additional item labeled “other,” which allows respondents to report traumatic events not covered by the previous categories. For each item, participants indicate their type of exposure using a 5-point response scale: *It happened to me; I witnessed it; I learned about it; It’s part of my job; or does not apply* (Blevins et al., 2015). The LEC-5 demonstrated good internal consistency in this study, with a Cronbach’s alpha of 0.77. The Portuguese validated version of the LEC-5 was administered alongside the PCL-5 to assess traumatic experiences corresponding to Criterion A for PTSD diagnosis.

Posttraumatic Stress Disorder Checklist for DSM-5 ((PCL-5) (Blevins et al., 2015; Weathers et al., 2013); Portuguese validated version (Carvalho et al., 2020), which involved translation and psychometric validation for the Portuguese population) is a 20-item self-report measure that assesses PTSD symptoms according to the four DSM-5 symptom clusters. Criterion A (exposure to traumatic events) is evaluated using the LEC-5. Items 1–5 correspond to Criterion B: Intrusion symptoms (e.g., “*Repeated, disturbing dreams about the stressful event*”); Items 6–7 to Criterion C: Avoidance (e.g., “*Avoiding memories, thoughts, or feelings related to the stressful event*”); Items 8–14 to Criterion D: Negative alterations in cognition and mood (e.g., “*Trouble remembering important parts of the stressful event*”); and Items 15–20 to Criterion E: Marked alterations in arousal and reactivity (e.g., “*Irritable behavior, angry outbursts, or acting aggressively*”). Participants rated how much they had been bothered by each symptom during the past month using a 5-point Likert scale ranging from 0 (*Not at all*) to 4 (*Extremely*). According to DSM-5 diagnostic guidelines (Ashbaugh et al., 2016), a provisional PTSD diagnosis requires a score of at least 2 (Moderately) on one item within each of the four symptom clusters, with total scores ranging from 0 to 80. Higher total scores indicate greater symptom severity. In the present study, the PCL-5 demonstrated excellent internal consistency (Cronbach’s α = 0.96), as well as high reliability across subscales: Intrusion (α = 0.93), Avoidance (α = 0.91), Negative alterations in cognition and mood (α = 0.92), and Arousal and reactivity (α = 0.88).

### 2.3. Procedure

This study was approved by the Ethics Committee of the University (name withheld for review) in March 2023. Subsequently, data collection followed from March 2023 to December 2023. Participants were recruited through convenience and snowball sampling methods. Study information and link to the questionnaire were distributed in social media platforms (Facebook and WhatsApp), and several institutional contacts were made, where two official European Portuguese entities—Associação Sindical dos Profissionais de Polícia (ASPP [Police Union Association]) and Guarda Nacional Republicana (GNR [National Republican Guard])—agreed to disseminate the study to their employees.

Prior to participation, all respondents were required to provide informed consent electronically. Participants could only proceed to the questionnaire after actively selecting the consent checkbox. No identifying or personally sensitive information was collected. An email contact for the principal investigator was provided for participants who wished to ask questions or seek clarification about the study. Completion of the questionnaire took approximately 15 min.

### 2.4. Statistical Analysis

Data analysis was performed using IBM SPSS Statistics version 28.0.0.0, employing both descriptive and inferential statistical methods. To examine the associations between traumatic events, the BSI dimensions, and PTSD diagnostic criteria, two Spearman correlation analyses were conducted. Correlation coefficients were interpreted as small (0.10–0.29), medium (0.30–0.49), and large (≥0.50) (Field, 2024). To compare the presence of psychopathological and PTSD symptoms between the general Portuguese community and first responders, four multivariate analyses of variance (MANOVAs) were conducted, with age included as a covariate in all analyses. Because the homogeneity of covariance matrices between groups was not met (*p* < 0.001), the MANOVA results were interpreted using Wilks’ Lambda *p*-values. Multiple linear regression analyses were then performed to determine whether the independent variables predicted the total PTSD Symptom Index. The independent variables were hierarchically entered in three blocks: (1) psychopathological variables: anxiety, depression, somatization, obsessive–compulsive symptoms, interpersonal sensitivity, hostility, phobic anxiety, paranoid ideation, and psychoticism; (2) trauma-related variables: traumatic events that happened to me, those that are part of my job, those I witnessed, and those I heard about; and (3) demographic variables: age, gender, education level, time in the current professional role, monthly household income, number of household members, health problems, medication use, and number of dependents. This blockwise hierarchical regression model was used to examine the proportion of variance explained by each block, given that the blocks contained variables of different typologies (e.g., traumatic experiences versus psychological symptomatology).

## 3. Results

### 3.1. Traumatic Events (General Community vs. First Responders)

Across the sample, exposure to a wide range of potentially traumatic events was reported. Among first responders (FR), 42.4% experienced a transportation accident personally, 36.0% had witnessed one, and 54.0% reported it as part of their occupational duties. Physical assault was similarly prevalent, with 42.0% reporting personal experience, 34.4% witnessing it, and 54.0% occupational exposure. Fire or explosions were witnessed by 41.4% and reported as occupational events by 54.0%. Sudden violent death and unexpected accidental death were frequently occupational, reported by 57.4% and 50.7% of FR, respectively. Sexual assault was personally experienced by 2.4% of FR, whereas other unwanted sexual experiences were reported by 6.1%. Less common events included captivity (0.6%) and combat-related exposure in war zones (10.9%). In the general community (GC), rates of personal trauma exposure were generally lower or comparable for natural disasters (16.1% FR vs. 15.3% GC), fire or explosions (13.7% FR vs. 15.3% GC), and transportation accidents (42.4% FR vs. 42.3% GC). However, GC participants reported higher rates of personal sexual assault (12.4%) and other unwanted sexual experiences (28.5%) compared to FR. Occupational exposure in the GC group was markedly lower across most categories, for example, work-related fire or explosion (5.1%), transportation accidents (4.4%), physical assault (7.3%), and sudden violent death (7.3%). Overall, FR reported substantially higher rates of occupational trauma across nearly all event categories, whereas the GC reported higher personal exposure for sexual and other unwanted sexual experiences.

### 3.2. Association Between Trauma Types, PTSD Symptoms, and Psychological Symptoms (BSI) in First Responders and the General Community

Regarding the correlations between trauma types, PTSD symptoms, and psychological symptoms (BSI) among first responders, work-related trauma showed small positive correlations with direct trauma (*r* = 0.155, *p* < 0.01), and moderate positive correlations with witnessed trauma (*r* = 0.423, *p* < 0.01), and heard-of trauma (*r* = 0.335, *p* < 0.01). However, work-related trauma was not significantly correlated with Criterion B, Criterion C, Phobic Anxiety, or Psychoticism symptoms. Direct trauma demonstrated small positive correlations with all PTSD Criteria and Psychological symptoms, ranging from r = 0.228 to r = 0.295 (*p* < 0.01), except for Phobic Anxiety and Psychoticism symptoms. Witnessed trauma was positively correlated with all variables (*r* = 0.155–0.384, *p* < 0.01). Heard-of trauma showed negligible to small correlations with Criterion C (*r* = 0.116, *p* < 0.01), Criterion D (*r* = 0.078, *p* < 0.05), Obsessive–Compulsive symptoms (*r* = 0.089, *p* < 0.05), Interpersonal Sensitivity symptoms (*r* = 0.101, *p* < 0.01), Depression symptoms (*r* = 0.097, *p* < 0.05), Hostility symptoms (*r* = 0.112, *p* < 0.01), and Paranoid Ideation symptoms (*r* = 0.136, *p* < 0.01). In the general community, work-related trauma was not significantly correlated with any Criteria or Psychological symptoms. Direct trauma showed small positive correlations with Criterion B (*r* = 0.273, *p* < 0.01), Criterion C (*r* = 0.287, *p* < 0.01), Criterion D (*r* = 0.275, *p* < 0.05), Criterion E (*r* = 0.305, *p* < 0.01), and with several Psychological symptoms, including Somatization symptoms (*r* = 0.271, *p* < 0.01), Depression symptoms (*r* = 0.186, *p* < 0.05), Anxiety symptoms (*r* = 0.210, *p* < 0.05), Hostility symptoms (*r* = 0.298, *p* < 0.01), Phobic Anxiety symptoms (*r* = 0.290, *p* < 0.01), Paranoid Ideation symptoms (*r* = 0.223, *p* < 0.01), and Psychoticism symptoms (*r* = 0.159, *p* < 0.01). Witnessed trauma in the general community did not show significant correlations with any PTSD Criteria or Psychological symptoms. Heard-of trauma was also not significantly correlated with any variables in this group. Finally, in both samples, the PTSD criteria were strongly intercorrelated (*r* = 0.533–0.777, *p* < 0.01 in FR; *r* = 0.506–0.777, *p* < 0.01 in GC) and showed moderate to strong positive correlations with Psychological symptoms (*r* = 0.366–0.846, *p* < 0.01 in FR; *r* = 0.366–0.780, *p* < 0.01 in GC).

### 3.3. Multivariate General Linear Model (GLM)

The first MANOVA (Table 2) compared the two groups (first responders and general community) based on four dependent variables (traumatic events that are part of my job; traumatic events that happened to me; traumatic events I witnessed; traumatic events I heard about), and revealed a significant effect of the independent variable on the variables traumatic events (that are part of my job, traumatic events I witnessed, and traumatic events I heard about). However, no significant effect was found for the variable of traumatic events that happened to me.

### 3.4. Presence of PTSD and Psychopathological Symptoms in Both Groups

The results of the second MANOVA (Table 3) showed a significant effect of the occupational variable for somatization, obsession-compulsion, interpersonal sensitivity, depression, anxiety, and psychoticism symptoms, but not for the hostility, phobic anxiety, and paranoid ideation symptoms, with age as a controlled covariate. In all cases where differences between groups were found, the general community had higher means than the first responders.

In the third MANOVA (Table 4), the variable Professional Activity did not have a significant effect on any of the variables: Criterion B (*p* = 0.472); Criterion C (*p* = 0.118); Criterion D (*p* = 0.175); and Criterion E (*p* = 0.478). The variable Age was tested as a covariate and did not show a significant effect on any of the variables: Criterion B (*p* = 0.234); Criterion C (*p* = 0.909); Criterion D (*p* = 0.227); and Criterion E (*p* = 0.831).

### 3.5. BSI Positive Symptoms and PTSD Positive Symptoms Index Among the General Community and First Responders

Multivariate analyses of variance were conducted to examine group differences between the General Community and First Responders in BSI Positive Symptom scores and PTSD Positive Symptom scores, while controlling for age as a covariate (Table 5). A significant main effect of group was observed for BSI Positive Symptoms, *F* (1,773) = 10.86, *p* < 0.001, partial η^2^ = 0.014. However, no significant group effect was found for PTSD Positive Symptoms, *F* (1,773) = 1.50, *p* = 0.22, partial η^2^ = 0.002.

### 3.6. Multiple Linear Regression—Predictors of PTSD Symptoms

Thus, the analysis resulted in a statistically significant model [*F* (13,740) = 98.193; *p* < 0.001; *R*^2^ = 0.633], including the variables from the first and second blocks, explaining 63% of the variance (Table 6). In the first block of variables, anxiety symptom (*β* = 0.901; *t* = 4.684; *p* < 0.001), depression symptoms (*β* = 0.457; *t* = 2.686; *p* = 0.007), obsessions-compulsions symptoms (*β* = 0.367; *t* = 2.511; *p* = 0.012), hostility symptoms (*β* = 0.445; *t* = 2.505; *p* = 0.012), paranoid ideation symptoms (*β* = 0.440; *t* = 2.965; *p* = 0.003), and psychoticism symptoms (*β* = 0.463; *t* = 2.052; *p* = 0.041) were found to be predictors of the total PTSD symptoms index. In the second block, only the variable traumatic events that happened to me (*β* = 0.582; *t* = 3.429; *p* < 0.001) was found to be a predictor of the total PTSD symptom index. The third block, which included demographic variables, did not significantly improve the model (*R*^2^ change = 0.007, *p* = 0.088), indicating that demographic factors did not contribute meaningfully to the prediction of PTSD symptoms in this sample.

### 3.7. ANOVA

A one-way ANOVA was conducted to examine whether marital status was associated with the total PTSD Symptom Index. The results indicated no significant differences between groups (*p* = 0.333).

## 4. Discussion

The first objective of this study was to examine the prevalence of exposure to traumatic events among first responders and the general community. The findings revealed that a substantial proportion of participants (76.4% of the total sample; 75.4% of first responders and 81.0% of the general community) reported having personally experienced at least one traumatic event during their lifetime. This prevalence is consistent with large-scale epidemiological research indicating that exposure to potentially traumatic events is widespread across populations, with approximately 70–75% of individuals reporting at least one such experience (Knipscheer et al., 2020). Similarly, national data from the Portuguese population have shown that nearly 69% of adults have encountered at least one traumatic event (Cardoso et al., 2020).

Consistent with previous research, the present findings confirmed that first responders are exposed to a greater number of traumatic events (Foley & Massey, 2021; Johnson et al., 2005; Lentz et al., 2021) than members of the general community (Knipscheer et al., 2020). When considering different forms of trauma exposure, such as the total number of traumatic experiences, events witnessed, those heard about, and particularly those occurring as part of occupational duties, first responders reported significantly higher exposure across all categories. However, no significant group differences were observed for personally experienced traumatic events.

The greater exposure of first responders to traumatic events can be attributed to the nature of their professional duties and the intense emotional demands inherent in their daily work. Their roles frequently involve high-risk physical tasks, such as detaining suspects, engaging in foot or vehicle pursuits, and responding to diverse emergency calls, that may pose threats to their own safety or that of others (Anderson et al., 2001). Although not all critical incidents are formally categorized as traumatic events (Diagnostic, 2013), the operational contexts in which these professionals perform their duties often expose them to situations that meet the criteria for potentially traumatic experiences (Carleton et al., 2018).

Regarding the second objective, based on the observed correlations, it appears that traumatic events among first responders are more associated with the development of psychopathological symptoms and PTSD compared to the general community. This is evidenced by significant correlations between nearly all trauma-related variables and the dimensions assessed by the PCL-5 and BSI instruments in the first responders’ group. In contrast, within the general community, only the variable *direct trauma* was associated with PTSD and psychopathological symptoms.

As previously discussed, due to the nature of their profession and responsibilities, first responders are exposed to traumatic events daily (Foley & Massey, 2021; Johnson et al., 2005; Lentz et al., 2021). Because their duties involve confronting danger and managing critical incidents, these professionals tend to maintain heightened vigilance and attribute greater significance to events they directly witness, hear about from others, or otherwise encounter indirectly, as such experiences are inherently tied to their occupational context. Exposure to traumatic events has been consistently associated with adverse mental health outcomes in prior research (Li et al., 2016), and specifically with PTSD symptoms among emergency medical professionals (Petrie et al., 2018). In the present study, and consistent with previous findings, first responders exhibited small but statistically significant positive correlations between several trauma exposure variables, namely, events that happened to me, those I witnessed, and those I heard about, and the PTSD diagnostic Criteria B, C, D, and E. However, events that are part of my job were correlated only with Criteria C and E, corresponding to avoidance and alterations in arousal and reactivity, which are particularly relevant to the performance demands of these professions.

Previous research has shown that professionals such as police officers and firefighters often perceive unexpected or uncontrollable events, particularly those involving life-threatening situations, as more distressing and threatening (Bryant & Harvey, 1996; Colwell et al., 2011). These findings reinforce the view that exposure to traumatic events is closely linked to the development of mental health difficulties, including PTSD, depression, and anxiety (Campillo-Cruz et al., 2021). Accordingly, the literature suggests that individuals with higher levels of trauma exposure are at greater risk of developing PTSD symptoms (Nourry et al., 2023).

In relation to the third objective, and based on prior research, it was expected that first responders would exhibit higher levels of psychopathological symptoms than the general community. Although significant group differences were observed, the results did not confirm this hypothesis. First responders reported a higher number of traumatic events overall; however, statistically significant differences emerged in six BSI dimensions: somatization, obsessive–compulsive symptoms, interpersonal sensitivity, depression, anxiety, and psychoticism, with the general community showing higher mean scores across all nine dimensions. Similarly, the general community also reported a greater total number of positive symptoms (BSI Positive Symptoms Index), indicating overall higher psychological distress compared to first responders.

A few studies are consistent with the present findings, indicating that the general community tends to exhibit higher levels of psychological distress compared to first responders (Goodwin et al., 2013). For instance, a study conducted in Wales, UK, during the COVID-19 pandemic found that first responders reported lower stress levels than the general population (Pink et al., 2021). Similarly, a Portuguese study involving a sample of 78 police officers reported levels of anxiety and depression comparable to those observed in the general population (Queirós et al., 2020).

Findings from these studies suggest that, although first responders are exposed to a greater number of traumatic events in their daily work, they appear to be less affected than the general community, reporting lower levels of depression and anxiety symptoms. This relative resilience may be attributed to professional training, higher levels of self-efficacy and self-esteem, occupational experience, and cultural narratives such as the “hero lifestyle,” which valorizes self-sacrifice in the service of others.

From a theoretical perspective, Terror Management Theory (TMT) (Solomon et al., 1991) provides an additional explanatory framework. TMT posits that although death is inevitable and unpredictable, individuals can buffer existential anxiety by constructing meaning and control through their actions and worldviews. In this context, first responders acknowledge the inherent dangers of their work but develop decision-making strategies aimed at preserving both their own lives and those of others (Rodriguez et al., 2016). They rely on their training, the competence of their peers, and the overarching sense of mission that helps them normalize and manage critical situations.

Moreover, repeated exposure to traumatic events may serve as a protective mechanism, fostering desensitization, adaptive learning from previous experiences, and more effective coping responses to subsequent stress and trauma (Burke & Shakespeare-Finch, 2011). In addition, organizational and cultural narratives surrounding first responder work, such as the ideals of bravery, service, and sacrifice, may contribute to a strong sense of purpose and collective identity (Rees et al., 2022). However, these same narratives, often aligned with military metanarratives of duty and the “warrior ethos,” can also reinforce organizational cultures of learned stoicism, where emotional restraint and endurance are valorized, and expressions of distress or help-seeking are implicitly discouraged (Heward et al., 2024; McElheran et al., 2024). At the personal level, trauma narratives of first responders often take the form of “we-narratives,” emphasizing collective identity, duty, and shared purpose. This collective framing serves as a form of meaning-making and identity maintenance that helps preserve psychological functioning and coherence when facing repeated exposure to stress and threat (Charretier et al., 2024). Distinguishing between adaptive stoicism and maladaptive emotional suppression may therefore be crucial to understanding the psychological profiles observed in these populations.

With regard to the fourth objective, which examined group differences in the diagnostic criteria for PTSD, it was anticipated that first responders would display higher symptom levels than the general community. However, despite their greater exposure to traumatic events and the significant correlations observed between trauma exposure and PTSD symptoms, no meaningful group differences were identified. None of the DSM-5 PTSD diagnostic criteria nor the PTSD Positive Symptom Index differed significantly between the two groups. These findings contrast with previous studies reporting higher prevalence rates of PTSD symptoms among first responders compared to the general population (Foley & Massey, 2021; Heir et al., 2019).

Several factors may explain the lower levels of anxiety and depression observed among first responders, as well as the absence of significant differences in PTSD symptoms between them and the general community. These factors include selection processes, professional training, social desirability bias, and the influence of “police culture” (Ostapovich et al., 2020). As previously noted, first responders are continually exposed to emotional strain arising from stressful and potentially traumatic situations. The selection processes for these professions likely play a crucial role in identifying candidates with leadership qualities, strong stress resilience, risk tolerance, high motivation, and a strong sense of self-efficacy, traits considered essential for performing such complex tasks (Ostapovich et al., 2020). Similarly, professional training may serve as a mitigating factor in the development of PTSD and other mental health conditions, which may help explain why, despite their greater exposure to traumatic events, first responders do not significantly differ from the general community (Sørensen et al., 2022).

The “police culture” is another critical factor shaped by the nature of police work, which involves social regulation and authority and is governed by informal rules and norms derived from occupational circumstances. This culture encompasses positive qualities such as support, teamwork, empathy, perseverance, and camaraderie but also promotes distrust, hypervigilance, and social isolation, which may contribute to reluctance in seeking help (Foley & Massey, 2021). Police culture can influence outcomes in two main ways (Hakik & Langlois, 2020; Westmarland, 2016). First, it may negatively affect research findings, as individuals could modify their responses due to concerns about confidentiality or a desire to avoid appearing vulnerable, thereby responding in a socially desirable manner (i.e., conforming to the societal expectation that weakness is incompatible with these professions) (Parkes et al., 2019a). Second, police culture may also exert a protective influence by fostering an “esprit de corps,” characterized by camaraderie, loyalty, and belonging, which strengthens perceived social support and group cohesion (Parkes et al., 2019b). Although the first responder sample in this study was not composed exclusively of police officers, these findings may not be fully explained by police culture alone. Nevertheless, the high proportion of police officers among participants, together with the overlapping occupational roles and shared professional characteristics across police, firefighters, and emergency medical personnel, suggests that cultural factors common to first responder professions should not be overlooked.

The results of this study indicated relatively high PTSD rates in both groups, 15.2% among first responders and 13.1% in the general community, when compared to findings from studies with similar samples. Following the Paris attacks in November 2015, for instance, PTSD prevalence was reported at 3.4% among firefighters and 9.5% among police officers (Motreff et al., 2020), contrasting with lower rates in community samples: 1.5% among adolescents aged 11–19 (Redican et al., 2022), and 3.9% (5.6% among those exposed to trauma) among adults over 18 years old (Koenen et al., 2017). However, other investigations have reported prevalence values similar to those observed in the present study, including PTSD symptoms in 10–15% of first responders (Klimley et al., 2018), 14.87% among police officers, and 8% in the general population in Canada (Wagner et al., 2020), and 11.1% in civilian populations more broadly (Spottswood et al., 2017).

PTSD symptoms have been linked to multiple psychological and contextual factors over time. The fifth and final objective of this study was to identify the factors associated with the presence of these symptoms in both samples. The findings indicated that anxiety, depression, obsessive–compulsive tendencies, hostility, paranoid ideation, and psychoticism were significant predictors of PTSD symptoms. Previous research has consistently shown that anxiety and depression exhibit high comorbidity with PTSD (Hyland et al., 2021; Karatzias et al., 2020) and often act as strong predictors, as observed in a study of 638 armed forces veterans in Ireland (Spikol et al., 2022). Regarding the broader psychopathological dimensions assessed by the BSI (in conjunction with the DASS-21, which together formed a single factor), a study of Portuguese firefighters similarly reported significant associations with PTSD symptoms (Becker et al., 2023). Comparable results have also been observed in general population samples. For instance, a study of 7403 individuals from the UK population (Gradus et al., 2013) found that PTSD symptoms frequently co-occurred with depression, substance use disorders, and various anxiety disorders (Qassem et al., 2021).

Consistent with previous studies (Becker et al., 2023)**,** the present findings confirmed that exposure to traumatic events was associated with the development of PTSD symptoms. However, among the different types of exposure examined, only the dimension referring to direct trauma (“traumatic events that happened to me”) emerged as a significant predictor of PTSD symptom severity. Recent research has increasingly focused on understanding not only the types of traumatic events (e.g., accidents, death of others, natural disasters) but also their cumulative impact and qualitative characteristics (Kubat & Duval, 2019). The present findings highlight that, across both samples, only direct exposure to traumatic events (“traumatic events that happened to me”) was a significant predictor of total PTSD symptom severity, while witnessed, work-related, and heard-of trauma did not show predictive value. This pattern underscores the centrality of direct exposure in the development of posttraumatic symptomatology. Although the DSM-5 expanded Criterion A to include indirect exposure, such as learning or hearing that a traumatic event occurred to a close family member or friend, our data indicate that this form of exposure was only weakly associated with specific PTSD symptom clusters (Criteria C and D) and with a limited range of general psychological symptoms. In first responders, “heard-of” trauma correlated moderately with other trauma types, suggesting that individuals frequently exposed to trauma may also report indirect exposure; however, its associations with PTSD clusters were weak and limited to Criteria C and D. In the general community, no significant correlations were observed. These findings contribute to the ongoing discussion about the conceptual boundaries of Criterion A in DSM-5. Consistent with evidence from a systematic review (May & Wisco, 2016), indirect exposure can elicit posttraumatic stress symptoms, but the likelihood and severity of these reactions are substantially lower than those observed after direct exposure. That review also emphasized the role of proximity as a determinant of PTSD risk, with indirect or distal experiences typically generating less intense and less enduring symptomatology. Together, our findings suggest that indirect exposure, as operationalized in the LEC-5, may represent a qualitatively distinct and less pathogenic form of traumatic experience, reinforcing the need for future research to refine Criterion A boundaries and to differentiate vicarious distress from full PTSD (Marx et al., 2024).

Demographic variables were examined to determine whether they predicted PTSD symptoms; however, none emerged as significant predictors, which contrasts with some previous findings (Schein et al., 2021). Although the present study did not reveal gender differences, previous research has consistently reported disparities between men and women, with women showing higher PTSD prevalence rates (e.g., 6.3% in women versus 3.9% in men) (Hyland et al., 2021). Similarly, a systematic review on PTSD prevalence in the United States found that, despite inconsistencies due to methodological, design, and sample differences, PTSD prevalence is generally higher among first responders, refugees, individuals with substance use disorders, transgender men, women, and younger adults (Schein et al., 2021). Beyond PTSD, a large-scale Canadian study involving 5813 first responders found that younger individuals, those with fewer years of service, individuals who were single, separated, divorced, or widowed, as well as women and those with higher education levels, exhibited a higher prevalence of mental health disorders overall (Carleton et al., 2018).

This study has some limitations that should be considered when interpreting the findings. The cross-sectional design prevents the establishment of causal relationships between exposure to traumatic events and PTSD symptoms, and future longitudinal studies are needed to clarify the temporal sequence of these associations. Moreover, the data were obtained through self-report measures administered in an online format, which, although practical for large and diverse samples, may be subject to self-selection bias, social desirability, and limited control over response conditions. In addition, given the organizational culture and professional norms characteristic of first responders, it is possible that some participants underreported symptoms or distress due to concerns about stigma or potential organizational repercussions. This tendency toward underreporting may have led to conservative estimates of symptom severity within this group. Another important limitation concerns the absence of a clinician-administered diagnostic interview, such as the Structured Clinical Interview for DSM-5 (SCID-5) or the Clinician-Administered PTSD Scale for DSM-5 (CAPS-5). Although the use of validated self-report instruments (LEC-5 and PCL-5) provided reliable symptom assessment, these tools do not replace a structured clinical evaluation conducted by trained professionals. Consequently, the prevalence and severity of PTSD symptoms reported here should be interpreted with caution. Finally, the non-probabilistic sampling limits the generalizability of the results to the broader populations of first responders and community members. Future research should consider combining self-report and clinician-administered tools, using probabilistic sampling methods, and employing longitudinal designs to enhance diagnostic precision and causal inference.

## 5. Conclusions

First responders are exposed daily to a greater number of traumatic events compared to the general community. The present study demonstrated that individuals with higher exposure to such events do not necessarily exhibit higher levels of psychopathology or PTSD symptoms. Furthermore, no significant associations were found between demographic variables (e.g., age, gender, or economic status) and the development of PTSD symptoms, suggesting that other mechanisms may underlie the emergence of these symptoms.

Although the findings align with previous research, they should be interpreted with caution, as methodological variations across studies, such as sample composition, geographic context, and instruments used to assess traumatic exposure, may influence the comparability of results (Benjet et al., 2016; Knipscheer et al., 2020). In this study, the wide age range of participants and the predominance of first responders (Angehrn et al., 2020a, 2020b) may also have influenced the outcomes.

Future research should explore factors such as terror management theory, training and selection processes, the “hero lifestyle,” and police culture to better understand why first responders exhibit levels of psychopathology and PTSD comparable to those of the general community despite their higher exposure to trauma.

The current findings, showing concerning levels of psychopathological symptoms and PTSD indicators in both groups, highlight the urgent need for identification, treatment, and prevention efforts targeting these populations. Culturally adapting and validating psychoeducational tools and interventions aimed at enhancing resilience, training, and education among first responders may be crucial. Moreover, public awareness campaigns should encourage help-seeking behaviors, particularly among first responders, to reduce stigma and promote mental health support within these professional groups.

## Figures and Tables

**Table 1:** Sociodemographic Characteristics.

Groups		*n* (%) or *M* (*SD*)
First responders		672 (83.1)
General community		137 (16.9)
Age	First responders	44.00 (8.62)
	General community	43.78 (10.87)
Gender	First responders	
	Male	585 (87.1)
	Female	87 (12.9)
	General community	
	Male	45 (32.8)
	Female	92 (67.2)
Marital status	Single	173 (21.4)
	Married	413 (51.1)
	Cohabitating	138 (17.1)
	Divorced or separated	84 (10.4)
	Widowed	1 (0.1)
Nationality	Brazilian	5 (0.6)
	Spanish	2 (0.2)
	Portuguese	800 (98.9)
	Swiss	1 (0.1)
	Venezuelan	1 (0.1)
Education level	Primary education	88 (10.9)
	Secondary education	500 (61.8)
	Bachelor’s degree	142 (17.6)
	Master’s degree	75 (9.3)
	Doctorate	3 (0.4)
	Post-doctorate	1 (0.1)
Health problems	Yes	185 (22,9)
	No	624 (77.1)
Medication	Yes	269 (33.3)
	No	525 (64.9)
PTSD criteria	First responders	102 (15.2)
	General community	18 (13.1)

**Table 2:** Traumatic events in the Professional Activity groups (First Responders and General Community).

	Professional Activity	*M* (*SD*)	Mean Square	*F*	Sig	Partial η^2^
Traumatic events that are part of my job			3360.01	142.87	<0.001	0.151
	FR	6.39 (5.17)				
	GC	0.96 (2.76)				
	Total	5.47 (5.26)				
Traumatic events that happened to me			5.558	1.122	0.290	0.001
	FR	2.39 (2.21)				
	GC	2.61 (2.32)				
	Total	2.47 (2.23)				
Traumatic events I witnessed			126.625	10.989	<0.001	0.013
	FR	3.30 (3.57)				
	GC	2.25 (2.30)				
	Total	3.12 (3.41)				
Traumatic events I heard about			101.239	4.691	0.031	0.006
	FR	4.43 (4.80)				
	GC	3.48 (3.81)				
	Total	4.28 (4.66)				

Note. FR = First responders (*n* = 672); GC = General community (*n* = 137).

**Table 3:** BSI dimensions in the Professional Activity groups (First Responders and General Community).

	Professional Activity	*M* (*SD*)	Mean Square	*F*	Sig	Partial η^2^
Somatization			108.77	6.75	0.010	0.008
	FR	3.51 (4.12)				
	GC	4.50 (3.65)				
	Total	3.68 (4.06)				
Obsessive–Compulsive symptoms			106.27	6.09	0.014	0.008
	FR	6.31 (4.15)				
	GC	7.27 (4.31)				
	Total	6.47 (4.19)				
Interpersonal Sensitivity symptoms			62.96	7.66	0.006	0.009
	FR	3.32 (2.84)				
	GC	4.06 (3.00)				
	Total	3.44 (2.88)				
Depression symptoms			143.10	7.73	0.006	0.010
	FR	4.73 (4.26)				
	GC	5.85 (4.51)				
	Total	4.92 (4.32)				
Anxiety symptoms			108.80	7.40	0.007	0.009
	FR	4.28 (3.79)				
	GC	5.26 (4.04)				
	Total	4.44 (3.85)				
Hostility symptoms			2.88	0.28	0.598	0.000
	FR	4.20 (3.29)				
	GC	4.35 (2.80)				
	Total	4.22 (3.21)				
Phobic Anxiety symptoms			5.19	0.78	0.376	0.001
	FR	1.65 (2.58)				
	GC	1.87 (2.56)				
	Total	1.70 (2.57)				
Paranoid Ideation symptoms			21.48	1.56	0.212	0.002
	FR	5.85 (3.70)				
	GC	6.28 (3.74)				
	Total	5.92 (3.72)				
Psychoticism symptoms			90.91	9.25	0.002	0.011
	FR	2.80 (3.11)				
	GC	3.69 (3.30)				
	Total	2.95 (3.16)				

Note. FR = First responders (*n* = 672); GC = General community (*n* = 137); BSI = Brief Symptom Inventory.

**Table 4:** Comparisons of the severity of self-reported diagnostic symptoms of PTSD among First Responders and in the General Community.

	Professional Activity	*M* (*SD*)	Mean Square	*F*	Sig	Partial η^2^
Criterion B			1.369	0.518	0.472	0.001
	FR	1.02 (1.63)				
	GC	1.13 (1.60)				
	Total	1.04 (1.63)				
Criterion C			1.707	2.444	0.118	0.003
	FR	0.52 (0.82)				
	GC	0.65 (0.89)				
	Total	0.54 (0.84)				
Criterion D			7.032	1.845	0.175	0.002
	FR	1.18 (1.93)				
	GC	1.43 (2.08)				
	Total	1.22 (1.95)				
Criterion E			1.548	0.503	0.478	0.001
	FR	1.44 (1.79)				
	GC	1.32 (1.57)				
	Total	1.42 (1.75)				

Note. FR = First responders (*n* = 672); GC = General community (*n* = 137); Criterion B = Intrusive symptoms; Criterion C = Avoidance; Criterion D = Negative alterations in cognition and mood; Criterion E = Marked alterations in arousal and reactivity.

**Table 5:** BSI positive symptoms, PTSD positive symptoms index, and Traumas according to professional activity groups (First Responders and General Community).

	Professional Activity	*M* (*SD*)	Mean Square	*F*	Sig	Partial η^2^
BSI positive symptoms			1.692	10.858	0.001	0.014
	FR	1.40 (0.39)				
	GC	1.52 (0.43)				
	Total	1.42 (0.40)				
PTSD positive symptoms index			339.328	1.502	0.221	0.002
	FR	35.40 (14.87)				
	GC	37.18 (15.76)				
	Total	35.70 (15.02)				

Note. FR = First responders; GC = General community; BSI = Brief Symptom Inventory.

**Table 6:** Hierarchical Regression Analysis of Predictors of the Total Index of PTSD Symptoms.

	B	*β*	*t*	*R* ^2^	*R* ^2^ ^(adjust)^
Predictors				0.633	0.627
Block 1				0.620	0.616
Anxiety	0.901	0.233 ***	4.684		
Depression	0.457	0.133 **	2.686		
Somatization	0.171	0.046	1.201		
Obsessions-Compulsions	0.367	0.102 *	2.511		
Interpersonal Sensitivity	0.000	0.000	−0.002		
Hostility	0.445	0.096 *	2.505		
Phobic Anxiety	0.251	0.043	1.190		
Paranoid Ideation	0.440	0.109 **	1.190		
Psychoticism	0.463	0.098 *	2.052		
Block 2				0.633	0.627
TE that happened to me	0.582	0.086 ***	3.429		
TE that are part of my job	−0.092	−0.032	−1.283		
TE I witnessed	0.223	0.051	1.848		
TE I heard about	0.079	0.024	0.968		
Block 3				0.640	0.630
Age	−0.073	−0.43	−1.144		
Gender	−0.206	−0.55 *	−2.191		
Education level	0.489	0.026	1.077		
Time in the current professional position	0.078	0.048	1.294		
Monthly household income	−0.430	−0.40	−1.536		
Number of household members	−0.714	−0.057	−1.560		
Health problems	0.852	0.024	0.887		
Medication use	−0.759	−0.024	−0.839		
Number of dependents	0.818	0.051	1.499		

Note. *** = *p* < 0.001; ** = *p* < 0.01; * = *p* < 0.05; TE = Traumatic events.

## Data Availability

The data that support the findings of this study are not publicly available due to institutional and confidentiality restrictions, but are available from the corresponding author upon reasonable request.

## Abbreviations

The following abbreviations are used in this manuscript:

PTSD	Post-Traumatic Stress Disorder
BSI	Brief Symptom Inventory
COVID	Coronavirus Disease
LEC	Life Events Checklist
GNR	Guarda Nacional Republicana
DSM	Diagnostic and Statistical Manual of Mental Disorders
PCL	Posttraumatic Stress Disorder Checklist
MANOVA	Multivariate Analysis of Variance
ASPP	Associação Sindical dos Profissionais de Polícia
SPSS	Statistical Package for the Social Sciences
TMT	Terror Management Theory
